# Families of Children/Youth with Complex Needs Before, During, and After COVID-19 Pandemic Restrictions

**DOI:** 10.1007/s40653-024-00676-9

**Published:** 2025-01-03

**Authors:** Kim Arbeau, Serena Atallah, Jeff St. Pierre

**Affiliations:** 1 Applied Research and Evaluation Department, Child and Parent Resource Institute, 600 Sanatorium Road, London, ON N6H 3W7 Canada; 2 Department of Psychology, Social Science Centre, Western University, London, ON N6A 5C2 Canada

**Keywords:** Families, Complex needs, Mental health, COVID-19, Pre-pandemic, Post-pandemic

## Abstract

During the COVID-19 pandemic, families with children who had complex special needs faced many obstacles and had less resources available to them. Little published research has described post-lockdown family functioning in clinical samples. The current study investigated caregiver well-being, family functioning, and child/youth symptomatology in three Canadian samples of families with children/youth who had pre-existing, complex, emotional, behavioural, developmental, and mental health needs pre-pandemic (clinic intake within 1 year prior to March 2020), COVID pandemic (clinic intake 1 year during societal lockdowns), and post-restrictions (clinic intake between summer 2022 and summer 2023). Cross-sectional archival data (*n* > 300 in each cohort) were compared from assessments completed by families as standard of care at a tertiary children’s service agency. As predicted, the pandemic intake sample reported significantly more parental mental health challenges, stress, conflicts, and went on fewer recreational outings than the pre-pandemic sample. While parent stress levels and family outing rates in the post-restriction sample resembled the pre-pandemic sample, post-lockdown parental mental health symptoms, family conflict, and family respite levels indicate that families are still recovering. Child symptom levels were high in all three samples, likely a result of intake criteria at this tertiary mental health agency. Research recommendations are offered. Clinical agencies should be mindful that some families of children with complex needs may still be impacted by the COVID-19 pandemic changes and consider using a family oriented, trauma-informed care approach to assess the effect of the pandemic.

When coronavirus disease 2019 (COVID-19) struck, families were suddenly faced with navigating a highly infectious virus and the restrictive measures intended to reduce the transmission of this life-threatening illness. Public health “stay in shelter” measures left some parents mentally and physically drained (e.g., Cluver et al., 2021; Kong, 2021; Lee et al., 2021; Marchetti et al., 2020; Nielsen, 2021). Parents variously worked from home, lost their jobs, worked extra hours as essential employees, became sick and self-isolated – while supporting children suddenly home full time. On March 11th, 2020, COVID-19 was declared a pandemic by the World Health Organization (WHO, 2020) and school closures were seen globally with an estimated 91% of students not attending classes in-person during the initial outbreak, equating to about 1.57 billion students not at school (Badr et al., 2020; Lee, 2020; UNICEF, 2020). Families weathered many months of uncertainty, not knowing when life would return to “normal” (Cluver et al., 2021; Gassman-Pines et al., 2020). In Ontario, Canada, schools closed for the first time in March 2020 (Freeman, 2023; Nielsen, 2021) and remained closed for six months before reopening with new protocols in September 2020 (e.g., masks on, extracurriculars cancelled, new virtual learning options; Elliott, 2022; Nielsen, 2021). Schools closed again in January 2021, April 2021, and January 2022 as the virus surged in Ontario, leaving parents and students with little consistency.

## Parenting in a Pandemic

Among adults participating in large-scale pandemic surveys across the world during the spring 2020 lockdown, parents/caregivers were especially likely to report stress and exhaustion. These surveys offer rich insight into pandemic coping. A large Canadian multi-informant survey in the spring of 2022 (Parsons Leigh et al., 2023) asked both parents and their child/youth, “compared to the time before the COVID-19 pandemic, how is your mental health”. Less than half of those dyads surveyed reported worsening mental health, indicating a general finding of resilience often noted in the pandemic population survey research literature. Their results do indicate that significantly more parents than children/youth reported a worsening of internalizing symptoms and fatigue since the pandemic began. In an Australian study, Westrupp et al. (2023) found that compared to pre-pandemic data, the pandemic elicited higher levels of parental stress, anxiety and depression, more irritability, partner conflict, and more alcohol consumption, with parents who had pre-existing physical/mental health challenges at greater risk. Parents surveyed have reported stress due to factors such as managing online learning, working from home, financial challenges, increased parental responsibility, increased psychological burden, fears of contracting COVID-19, navigating illness prevention protocols when parents lived apart, potential for conflict in the home, self-isolation, and concern about the mental health impacts of the pandemic on their children (American Psychological Association, 2020a; American Psychological Association, 2020b; Bentenuto et al., 2021; Brown et al., 2020; Gadermann et al., 2021; Giannotti et al., 2022; Goldberg et al., 2021; Nair et al., 2024; Painter et al., 2024; Pierce et al., 2020; Spinelli, Lionetti, Pastore et al., 2020; Spinelli, Lionetti, Setti et al., 2020). When interpreting these large population surveys of pandemic family life, sample bias concerns should be noted; many studies are crowdsourced online, while others specify random selection and probability sampling using detailed polling parameters (e.g., Patrick et al., 2020).

Parenting was identified as a moderating factor in worsening mental health by Gadermann and colleagues (2021) with 44% of parents reporting deteriorated mental health due to the pandemic versus 36% of adults without children under age 18. Again, we note the majority did not report a deterioration, and it must be emphasized that despite many new stressors, both decline and resilience in parental mental health are seen in this pandemic literature (Bentenuto et al., 2021; Brown et al., 2020; Feinberg et al., 2022; Findlay & Arim, 2020; Gayatri & Irawaty, 2022; Lee et al., 2021; Thomson et al., 2021; Verdolini et al., 2021). Some parents reported enjoying connections within the family and a less hectic schedule during the pandemic (Visone et al., 2022). Parent/child co-participation in exercise was related to increased happiness, improved family relationships, and decreased loneliness during the pandemic (Koga et al., 2023). In a non-clinical sample, Prime and colleagues (2022) identified four profiles of caregivers based on characteristics related to type of stress (i.e., income, family, pandemic-related) and adaptation during the pandemic. A minority of caregivers (7%) were reported to be managing multiple levels of stress/disruption, and this group had the least social support and greatest caregiver and child/youth mental health challenges. Most caregivers (54%) fell into the subgroup with low levels of stress/disruption, which had minor difficulties and reported positive impacts from the pandemic such as new routines and family connectedness. Essler et al. (2024) reported a steady decrease in family well-being in the first year of the pandemic, and a negative relation between parental stress and child psychological well-being as the pandemic progressed. One research group who extensively reviewed the literature on family and youth adjustment during the pandemic hypothesized that societal lockdowns may have served as an “enhancer” – family relationships that were already strong got better, while those already struggling got worse during isolation (Campione-Barr et al., 2024). This research points to the importance of understanding the etiology of the differential impacts of the pandemic on families.

## Impacts of the Pandemic in Families with Children and Youth with Special Needs

Children with special needs represent a unique subgroup of interest during times of rapid societal change and may account for some of the heterogeneity seen in reports on pandemic family coping (e.g., Latzer et al., 2021; Lipkin & Crepeau-Hobson, 2023). Cognizant of a diathesis-stress model of illness and coping, it is anticipated this societal crisis will disproportionately impact families with pre-existing mental health vulnerabilities (Campione-Barr et al., 2024; Prime et al., 2020, 2022). Parents of children with higher social-emotional-behavioural needs have reported relatively more stress and exhaustion during the pandemic (Bentenuto et al., 2021; Marchetti et al., 2020; Miniarikova et al., 2022; Skripkauskaite et al., 2023; Tso et al., 2020; Wang & Zhang et al., 2022). Willner et al. (2020) reported 43% of those caring for a child with an intellectual disability had moderate/severe anxiety and 31% had major depression, compared to 8% and 3% of caregivers, respectively, whose children did not have an intellectual disability. Parents caring for children with special educational needs (Zampini et al., 2024) and autism (Knedlíková et al., 2024) showed greater parental stress during the pandemic lockdown period compared to parents of typically developing children. Caretaking without breaks, supporting children’s education and recreation in the home, and monitoring time on electronic devices was challenging for parents of children with special needs (Adams et al., 2024; Colizzi et al., 2020; Isensee et al., 2022; Jacques et al., 2022; Lipkin & Crepeau-Hobson, 2023; Shorey et al., 2021), with some parents indicating it was difficult to explain the new changes and rules to their child (Wolstencroft et al., 2021). Increases in severity, intensity, and frequency of challenging behaviours were noted by parents of children with intellectual and developmental disabilities (Biggs et al., 2024; Shorey et al., 2021), neurodevelopmental disorders (Wolstencroft et al., 2021), and autism (Colizzi et al., 2020; Tawankanjanachot et al., 2024). A study from Germany found 16% of parents with children diagnosed with autism required an increase in their child’s dosage of medication during the pandemic (Isensee et al., 2022). Health concerns included sedentary behaviour, sleep challenges, and unhealthy eating (Shorey et al., 2021).

Consistent with social ecological theories suggesting an interactive relationship between a child’s behaviour and their family environment (e.g., Bronfenbrenner, 1979), pandemic research shows that parental well-being may have influenced and been impacted by their children’s degree of social-emotional-behavioural challenges. Achterberg et al. (2021) reported in a longitudinal analysis that pre-COVID parental stress predicted both acute lockdown mental health concerns in the parent and behaviour concerns in the child. As children with special needs generally tend to experience greater behavioural challenges (Baker et al., 2003; Hayes & Watson, 2013; Janus et al., 2018), pandemic changes to daily routines may have initiated a transactional process of deteriorating child and parent interactions. Evidence of increased conflict in the home between parents, children, and siblings during the pandemic has been noted for some families of children with special needs (e.g., Isensee et al., 2022; Toseeb, 2022).

### The Role of Formal and Informal Supports

Loss of supports was notable during the pandemic (e.g., Essler et al., 2024; Hawke et al., 2020; Patrick et al., 2020; Spinelli, Lionetti, Pastore et al., 2020). In our province (Ontario, Canada) the rates of girls ages 10 to 17 seeking emergency/acute care hospitals for suicidal concerns increased notably during the first 2 years of the pandemic (Mitchell et al., 2023), a possible result of loss of normal supports. During the pandemic, parents of children with special needs lost direct access to the specialized home, school, recreation and social services they relied upon, increasing their time as educators and therapists for their children (Arim et al., 2020; Bentenuto et al., 2021; Biggs et al., 2024; Colizzi et al., 2020; Fisher et al., 2020; Kong, 2021; Latzer et al., 2021; Ren et al., 2020; Shorey et al., 2021). Social and emotional support and respite, including unpaid help from family and friends, has been reported as valuable and anxiety-reducing for parents of children with special needs (MacDonald & Callery, 2004; Ren et al., 2020). In an online Canadian survey during the pandemic, parents of children with autism reported poignant experiences of isolation from extended family, loneliness, trying to support the child’s education while schools were closed, managing difficult child behaviours, and struggling to access informal and professional services and respite (Jacques et al., 2022). Parents of children with intellectual disabilities in the U.K. reported similar experiences (Willner, 2020; Wolstencroft, 2021).

### Resilience Factors in Special Needs Families

Although challenges were reported for families of children with special needs during lockdown, some families reported benefits including examining career options, slowing down, and understanding what is important in their lives; protective behaviours included creating a routine, positive communication between caregivers, spending quality time together, physical activity, and being able to advocate more for their child’s learning needs during at-home education (Biggs et al., 2024; Camargo et al., 2023; Isensee et al., 2022; Jacques et al., 2022; Shorey et al., 2021). Some reported their child’s pre-existing mental health concerns improved during pandemic restrictions, noting their child was happy with being at home and not being around other people (Wolstencroft et al., 2021). A study also indicated a decline in peer relationship difficulties in clinically referred children/youth during the pandemic as compared to before the pandemic (Manis & Stewart, 2024). Families of children with autism who noted benefits of the pandemic were found to report less mental health struggles in their child, higher self-efficacy, and a more positive family environment (Isensee et al., 2022).

### Longevity of Pandemic Effects

The reports reviewed above do not describe the long-term impacts of the pandemic and societal lockdown on families in the general population, nor those with a child with special needs. Acute impacts have been well studied in the general population in many countries and initial concerns of a corollary mental health pandemic have eased. In a large systematic review and meta-analysis of acute pandemic changes in mental health that included 137 investigations, Sun et al. (2023) reported no average change for men and very low magnitude average change for women on mental health repeated measures of symptoms in 2018–2019 versus 2020. Likewise, there were no indications of significant changes found across 16 cohorts of children/adolescents on repeated measures of general child mental health. While this resilience is noteworthy, “average change” masks the significant heterogeneity in coping across these studies. Of note, worsening of anxiety and mental health symptoms were more likely to be reported by adults who were parents.

In a more recent review that goes beyond the first wave of lockdowns, Orban et al. (2024) reviewed 24 investigations of child and youth mental health pandemic changes over time in general population studies. The quality of the research reviewed varied, and the findings were complex. Generally, Orban and colleagues concluded that there was a clear increase in the number of children and youth reporting internalizing symptoms during peak public health restrictions, and that these concerns had not fully resolved after most acute lockdowns lifted in 2021. Since that review, a large German longitudinal population survey of COVID-19 and youth psychological health (COPSY) has published results using self-report scales of good validity across five timepoints, from spring 2020 to fall 2022 (Ravens-Sieberer et al., 2023). They report that youth mental health underwent a deterioration in the first two years of the pandemic that is now improving as of fall 2022. They note that by the 2022 data collection, youth in Germany (now out of lockdown) were reporting new worries, for example the Ukraine war. The increased number of youth participants reporting increased depressive symptoms had returned to pre-pandemic levels, however some other symptom scores (e.g., youth psychosomatic symptoms) remained elevated in fall 2022.

In another German longitudinal population study, Benke et al. (2023) followed adults using online surveys administered in spring 2020, late 2020, and spring 2021. Again, the results are complex but generally increases in depression and loneliness were reported at one-year follow-up, especially for those with pre-existing mood concerns, even though COVID related stress improved. Camargo et al. (2023) reported (on 53 studies) in their review of pandemic depression symptoms during social lockdowns that females, young people, and those with pre-existing depression were at greater risk. They report conflicting results regarding symptom continuity as pandemic restrictions were eased. In a longitudinal non-clinical sample in Italy (parents undergoing psychiatric diagnosis/treatment or those who had a COVID infection were exempted), Cimino et al. (2024) completed repeated measures of a common adult symptom checklist and a child behaviour checklist in 2019, 2020, and 2021. They report that mothers not at-risk for mental health challenges pre-COVID reported increased depression and somatization symptoms during the pandemic lockdown in 2020, which decreased again in 2021. The authors do not describe how pandemic restrictions policies/procedures had eased in Italy in 2021. It is noteworthy also that the statistical increase in adult self-reported symptoms during the pandemic remained in the non-clinical range of scores. Interestingly, interpersonal sensibility, hostility, and anxiety decreased during lockdown and increased again (note still in non-clinical range) when restrictions lifted, suggesting that returning to regular routines may have been stressful for some mothers. For children of not at-risk mothers, their psychological risk increased from pre-pandemic to lockdown and decreased again in the post-lockdown period, according to parent report. For mothers self-reporting they were at-risk for mental health challenges pre-COVID, their report of symptoms surprisingly decreased from Time 1 to Time 2 to Time 3. The authors suggest this could be due to a positive “shelter effect” of the lockdown, where they felt protected from the dangers of the virus. For children of at-risk mothers, psychological risk decreased from the pre-pandemic to the lockdown period and increased when restrictions lifted, suggesting that the lockdown had a protective effect on this group of children.

Published repeated measures longitudinal investigations of family coping over the past four years are rare as of late 2024. Given that Orban et al. (2024) in their systematic review estimated that the number of youth reporting concerns with internalizing symptoms doubled in the first year of the pandemic lockdowns, follow-up data collected during the endemic (post-lockdown) stage of the virus is required to assess whether changes are ongoing. We found one large multiyear (four distinct surveys from late 2020 to spring 2023) cross-sectional probability sample population study that asked about family functioning across more than one wave of pandemic restrictions in Germany (Geprägs et al., 2024). This study repeats the general finding that most families have coped well the three years since COVID-19 was declared a pandemic. While the questions surveyed are brief, they have the advantage of asking parents specifically whether relationships in the home and quality of life were better or worse since the pandemic began. A large majority report no ongoing parent-child and marital relations concerns or quality of life concerns; three quarters of the parents surveyed reported no change in parent-child relations. In wave one (2020/2021) 10% of parents reported worsening relations with their child, this was down to 5% in the spring of 2023. Common risk factors such as lower household income and pre-existing mental health concerns did appear to help identify a small at-risk group.

In a qualitative study, Adams and colleagues (2024) interviewed caregivers of children with cerebral palsy to gather their experiences during and after the pandemic period. They found that caregivers experienced emotional challenges related to being under lockdown with their child which extended after lockdowns were lifted; namely, worrying about how their child’s activities during lockdown (i.e., sedentary lifestyle, time spent on electronics) may have negatively impacted them post-lockdown. Social isolation was another challenge that continued for some caregivers after the pandemic, who noted that people they used to see before lockdown no longer called to make plans. Financial strain was an ongoing burden, with some caregivers reporting that they lost their jobs during the pandemic and were still not able to find work post-lockdown. Despite these challenges that continued after pandemic lockdowns ended, caregivers still reported feeling positively after lockdowns lifted because they could take a break from their children and children did not depend solely on the caregivers for support. Qualitative studies of this nature glean rich data about the experiences of caregivers of children with special needs during and after pandemic lockdowns.

Coller and colleagues (2024) examined whether parents’ perceptions of school safety impacted school attendance for children with medical complexities before wave 3, during waves 3 and 4, and after wave 4. Harris and Konkel (2024) examined the relationship between neighbourhood factors and the location of family violence reported before, during, and after lockdowns. Using community samples and a longitudinal design across three timepoints (pre-pandemic, during initial stay-at-home orders, and 6 months after the initial stay-at-home order was lifted) Rodman and colleagues (2024) examined social factors that protected against increases in youth psychopathology during the pandemic while controlling for pre-pandemic symptoms. However, to our knowledge no study has examined family functioning and child symptomology for children and families with complex special needs using data from before, during, and after pandemic restrictions were eased. Two studies that began during lockdown reported specifically on parents of children with mental health needs at different time points of the pandemic. Vella Fondacaro and colleagues (2023) in Malta found in a repeated measures study (62% attrition rate, which they noted may impact the generalizability of their results) that high parental anxiety early in the pandemic decreased longitudinally as pandemic restrictions eased. In contrast, Wang and Zhang et al. (2022) in China found in a large cross-sectional study of parents with children diagnosed with autism that the parent cohort reporting in late 2021 showed the same or higher levels of anxiety and depression symptoms compared to the 2020 cohort. Importantly, Vella Fondacaro et al. (2023) and Wang and Zhang et al. (2022) used different timepoints, with the former examining how parents fared as restrictions eased, and the latter examining how parents fared after restrictions were eased but then put back in place (regionally) as the virus resurged. In terms of family functioning after schools reopened, Toseeb’s (2022) mixed repeated measures and cross-sectional study examining sibling conflict in the U.K. indicates the rate at which children with special needs were victimized by their siblings spiked during the first lockdown (early 2020) and declined when schools reopened (late 2020).

To summarize, a sweeping general population mental health deterioration from COVID-19 is not evident from the research methods used to date. There are indications that parents do appear more impacted by COVID related stressors, and children, youth and adults experiencing pre-existing mental health concerns appear to be more likely to evidence an impact from COVID societal changes. Research comparing pre-pandemic, pandemic, and post-restriction functioning in at-risk families is warranted.

## Objectives

Children who are considered to have complex needs, that is those with multiple, profound needs including behavioural, emotional, developmental, and mental health challenges that require extensive supports, and the parents who care for them, are among at-risk groups (Hill et al., 2014; McCann et al., 2012; Van den Steene et al., 2019). Research suggests that adults with pre-existing mental health concerns (Fancourt et al., 2021) and those caring for a vulnerable child with clinical needs do have a disproportionate burden in coping with COVID-19 changes (Marchetti et al., 2020; Skripkauskaite et al., 2023; Willner et al., 2020), however little is known about adjustment in the post-lockdown timeframe in these at-risk populations.

The present study investigates three cross-sectional clinical samples: before, during, and after pandemic restrictions aimed at reducing virus transmission were lifted. We hope to examine the possible impacts of the COVID-19 pandemic and social lockdown on those seeking clinical services for children/youth with complex emotional, behavioural, developmental, and mental health needs. The current study uses existing quantified intake data from a large children’s tertiary service agency. Parents from this same tertiary agency historically report very high child-related and personal stress levels (Noh et al., 1989). We investigate child/youth internalizing and externalizing behaviours and basic demographics to examine differences at referral across the independent time samples. Based on the available literature discussing pandemic experiences for families of children with special needs, we predict that families seeking help for children with complex needs will demonstrate higher levels of parental mental health challenges, caregiver stress, and family conflict, and lower levels of unpaid respite support and family outings during the pandemic compared to a similar cohort of children assessed at the same agency pre-pandemic. We compare a post-restrictions cohort to the pre-pandemic and pandemic groups to determine if reports differ after social restrictions and COVID-19 infections eased. We predict that there will be lower rates of caregiver stress and family conflict for the post-restrictions cohort compared to the pandemic cohort, and higher rates of family outings and respite in the post-restrictions period compared to the pandemic period, with levels equivalent to the pre-pandemic period. As for parental mental health, there is little post-COVID data to guide us. We speculate that mental health concerns exacerbated by COVID life changes have not dissipated quickly, especially in caregivers of children with high needs. Results will inform clinicians about the potential impacts COVID-19 may have had on their clients and families with complex needs and may also inform strategies to improve support in future crises.

## Method

In the current study, three unique samples of children/youth ages 4 to 18 who had initial intake assessments completed at a single tertiary agency were compared. Outpatient services requested at this tertiary agency are typically for assessment by a child psychiatrist, with many who are also seen by multidisciplinary teams including psychologists, social workers, speech language pathologists, pediatricians, child behaviour counsellors, etc. Typically, children and youth referred to this agency experience a combination of severe externalizing behaviour problems and internalizing symptoms, with family dysfunction, and have previously received psychotropic medication treatments and/or psychosocial and educational supports that often began at a young age. The referent is typically a front-line clinician/medical professional or educator who indicates the child/youth is not coping despite previous supports and treatments, and further understanding and support is requested by the family. Common referral concerns include impulsive and disruptive behaviour, anxiety, trauma, autism, and dual diagnoses (intellectual and mental health concerns). Post COVID-19 outpatient services at this agency were quickly transitioned to virtual services using computer streaming and telephone consults with clinicians, followed by a very slow reopening of face-to-face contact (which followed public safety protocols).

The first sample consisted of 374 children/youth between the ages of 4 to 18 (66.8% males, 32.9% females, 0.3% reported as not identifying as male or female; *M*_*ag*e_=11.02, *SD* = 3.42) who sought outpatient services for complex needs at a children’s tertiary service agency in Ontario, Canada within 12 months before the pandemic start date (March 1, 2019 to February 29, 2020; pre-pandemic sample). In this sample, 98.4% spoke English as their primary language. A relative or nonrelative other than the parent was the legal guardian for 4.8% and a child protection agency was the legal guardian for 2.1%.

The second sample included 371 children/youth from the same tertiary service agency (62.0% males, 35.3% females, 2.7% reported as not identifying as male or female; *M*_*age*_=11.19, *SD* = 3.29) who sought outpatient services from October 1, 2020 to September 30, 2021 (pandemic sample). During the pandemic lockdown outpatient services were provided virtually by this agency (Theall et al., 2023). We chose six months into the pandemic as the starting point for children/youth in the pandemic sample because of data availability (the agency switched to a shorter screening tool during the first six months of the pandemic to reduce family burden) and this also allowed time for pandemic-related restrictions to demonstrate impact on children/youth and families. Some studies have emerged indicating that the pandemic had increasing impacts on children and families as it continued (Essler et al., 2024; Larsen et al., 2023). Families in this second cohort had experienced school closures, work changes, social distancing, mask mandates, vivid news reports of rates of infection and deaths from COVID-19, and three waves of the pandemic in Ontario (Nielsen, 2021). English was the primary language spoken for 97%. A relative or nonrelative other than the parent was the legal guardian for 8.9% and a child protection agency was the legal guardian for 2.4%.

The third sample consisted of 303 children/youth receiving services from the same agency (58.1% males, 38.6% females, 3.3% reported as not identifying as male or female; *M*_*age*_=11.86, *SD* = 3.33) who sought outpatient services from July 1, 2022 to June 30, 2023 (post-restrictions sample). During this time in-person services were being offered by the agency, with remote services still available. We chose this one-year timeframe because school classrooms were open and during the summer of 2022 most official restrictions had been removed and most activities for children and families had resumed (see Ontario timeline by Freeman, 2023). English was the primary language spoken for 97.4%. A relative or nonrelative other than the parent was the legal guardian for 7.3% and a child protection agency was the legal guardian for 3.0%.

Data from this study were archival, deidentified, and the cases are unique across all samples. The cohort group demographics were similar due to the large sample sizes and agency criteria, although there was a significantly higher proportion of youth (ages 12 to 18) to children (ages 4 to 11) in the post-restrictions sample compared to the pre-COVID-19 sample (see Table 1). No attempt was made to match demographics between the cross-sectional samples. At this very large tertiary agency all referents met the same basic entry criteria for intake needs severity and had sought clinical/educational supports previously in their home region or county in Ontario. Ethics approval was obtained from the research ethics board at Western University in London, Ontario, Canada.

**Table 1:** Comparison of sample characteristics for each cohort using chi-square analyses

Characteristics	Pre-COVID Cohort Sample 1		COVID Restrictions Cohort Sample 2		Post-Restrictions Cohort Sample 3		Cohorts 1 & 2 Compared		Cohorts 2 & 3 Compared		Cohorts 1 & 3 Compared	
	*n*	%	*n*	%	*n*	%	χ^2^	*p*	χ^2^	*p*	χ^2^	*p*
Gender							.749^a^	0.387	1.123	0.570	3.149^a^	0.076
Male	250	66.8	230	62.0	176	58.1						
Female	123	32.9	131	35.3	117	38.6						
Other	< 10	0.3	10	2.7	10	3.3						
Age							0.381	0.537	3.489	0.062	6.372	0.012
4–11	203	54.3	192	51.8	134	44.2						
12–18	171	45.7	179	48.2	169	55.8						
Language							0.996	0.318	0.000	0.984	0.449	0.503
English	368	98.4	360	97.0	295	97.4						
Other	< 10	1.6	11	3.0	< 10	2.6						
Legal guardianship							3.775	0.052	0.108	0.743	1.928	0.165
Parent(s)	348	93.0	329	88.7	272	89.8						
Non-parent	26	7.0	42	11.3	31	10.2						

Note. ^a^ Indicates that there were less than 5 cases for “Other” so this group could not be included in the chi-square analysis

### Measures

Data for this study were collected from the following two instruments within the interRAI™ Child/Youth suite: Child and Youth Mental Health (ChYMH; Stewart, Hirdes et al., 2015), and Child and Youth Mental Health and Developmental Disability (ChYMH-DD; Stewart, LaRose et al., 2015). Instruments from the interRAI Child/Youth suite use common language and are designed to be integrated together to support the collection of comparable information and continuity of care across services from infancy onwards; the suite includes assessments for inpatient and community mental health/developmental disability settings as well as emergency departments, youth justice facilities, and schools (Stewart et al., 2022). The ChYMH and ChYMH-DD are standard of care at this tertiary agency. They are both comprehensive, multidisciplinary, and multi-sourced instruments that each include over 400 data elements. Trained assessors choose which of these two instruments is most appropriate for a client. The ChYMH is completed for children and youth who have mental health concerns (and who may have challenges in other areas like safety, life skills, school adjustment, etc.) and the ChYMH-DD includes the needs/symptoms indicated in the ChYMH as well as needs specific for children/youth with developmental disabilities. The tools include a variety of areas related to child/youth health and well-being, including mental state indicators, substance use, harm to self and others, behaviour symptoms, strengths and resilience, communication, independence in daily activities, family and social relations, and health conditions.

The ChYMH and ChYMH-DD are each completed by trained assessors who collect information through a semi-structured interview with the parents/guardians (as well as the child/youth if appropriate), and gathering information from other relevant sources (e.g., clinical reports, referral documents, other involved professionals). The interviews are approximately 60 to 90 minutes, and assessors code tool items and enter notes into a software system. The assessors complete three days of training, that includes guidance on how to interpret/embed the information into their clinical work and are provided with a comprehensive manual. Data are stored electronically with a number ID (separate from identifiers like client name and address). In the current study, items that focused on the family context and child internalizing and externalizing symptomology were relevant. The following items were included in this study: “parent/caregiver has current developmental or mental health issues” (coded as no or yes), “major life stressors for parent(s)/primary caregiver(s) in last 90 days” (coded as no or yes), “conflict with or repeated criticism of family” (recoded as no/not applicable or yes), “unpaid respite supports” (recoded as not needed/available or not available), and “family went on outings for recreation” (recoded as never/not within last 30 days or yes, within the last 30 days). Child internalizing (12 items, e.g., unrealistic fears, withdrawal from activities of interest, expressions of hopelessness) and externalizing (12 items, e.g., violence to others, physical abuse, bullying peers) scales were used to measure child symptomology. Cut-points for both scales were recoded to “no risk to moderate risk” and “high/very high risk.” A comorbid internalizing and externalizing measure was created to capture a group of children/youth who were high on both scales by coding children/youth who were “high/very high risk” for both internalizing and externalizing as 1 and all other children/youth as 0. Multiple studies have reported the instruments within the interRAI Child/Youth suite to have strong psychometric properties (e.g., Lau et al., 2019, 2021; Stewart & Hamza, 2017; Stewart et al., 2020, 2021, 2022), including inter-rater reliability (Stewart et al., 2022). The adult interRAI suite, in which the Child/Youth suite shares items, has also been shown to have acceptable inter-rater reliability (Hirdes et al., 2008). Scale cut-points have been created using Receiver Operating Characteristic (ROC) curve results (Fadiya, 2016, 2019). Lau et al. (2019, 2021) reported the internalizing and externalizing scales as having acceptable internal consistency and criterion validity results. In the present study, the Cronbach’s αs for the pre-pandemic, pandemic, and post-restrictions samples respectively were: internalizing scale 0.83, 0.81, and 0.81, and externalizing scale 0.85, 0.79, and 0.79.

### Analyses

Analyses were run using chi-square tests for independence (with Yates’ continuity correction). For each variable (parental mental health; caregiver stress; family conflict; unpaid respite; family outings; child/youth symptomology), comparisons were made between samples. The phi coefficient is reported to indicate the strength of the relationship between the variables. The phi coefficient can range from 0 to 1 and can be either positive or negative depending on the direction of the relationship (Pallant, 2020; Prematunga, 2012).

## Results

The following sections will describe the group comparisons. For a summary of the results see Table 2 - pre-pandemic sample compared to pandemic sample, Table 3 - pandemic sample compared to post-restrictions sample, and Table 4 - pre-pandemic sample compared to post-restrictions sample.

**Table 2:** Frequencies and Chi-Square results for comparisons between Pre-COVID-19 and during COVID-19 for Family related experiences

Family Related Experience	Within 12 months before the COVID-19 Pandemic		During 12 months within the COVID-19 Pandemic		χ^2^ (Phi φ)	*p*
	*n*	%	*n*	%		
Parent/caregiver has current developmental or mental health issues	186	49.7	220	59.3	6.49 (0.10)	0.011
Major life stressors for parent(s)/primary caregiver(s) in last 90 days	133	35.6	197	53.1	22.51 (0.18)	< 0.001
Conflict with or repeated criticism of family	63	16.8	88	23.7	5.03 (0.09)	0.025
Unpaid respite supports unavailable	112	29.9	135	36.4	3.20 (0.07)	0.074
Family went on outings for recreation	331	88.5	280	75.5	20.57 (-0.17)	< 0.001
Child internalizing symptoms	147	39.3	132	35.6	0.95 (-0.04)	0.330
Child externalizing symptoms	134	35.8	125	33.7	0.29 (-0.02)	0.592
Child comorbid internalizing and externalizing symptoms	72	19.3	42	11.3	8.44 (-0.11)	0.004

**Table 3:** Frequencies and Chi-Square results for comparisons between during COVID-19 and post-restrictions for Family related experiences

Family Related Experience	During 12 months within the COVID-19 Pandemic		During 12 months Post-Restrictions		χ^2^ (Phi φ)	*p*
	*n*	%	*n*	%		
Parent/caregiver has current developmental or mental health issues	220	59.3	178	58.7	0.00 (-0.01)	0.947
Major life stressors for parent(s)/primary caregiver(s) in last 90 days	197	53.1	114	37.6	15.46 (-0.15)	< 0.001
Conflict with or repeated criticism of family	88	23.7	69	22.8	0.04 (-0.01)	0.843
Unpaid respite supports unavailable	135	36.4	118	38.9	0.36 (0.03)	0.547
Family went on outings for recreation	280	75.5	277	91.4	28.47 (0.21)	< 0.001
Child internalizing symptoms	132	35.6	110	36.3	0.01 (0.01)	0.909
Child externalizing symptoms	125	33.7	101	33.3	0.00 (0.00)	0.987
Child comorbid internalizing and externalizing symptoms	42	11.3	45	14.9	1.55 (0.05)	0.213

**Table 4:** Frequencies and Chi-Square results for comparisons between Pre-COVID-19 and post-restrictions for Family related experiences

Family Related Experience	Within 12 months before the COVID-19 Pandemic		During 12 months Post-Restrictions		χ^2^ (Phi φ)	*p*
	*n*	%	*n*	%		
Parent/caregiver has current developmental or mental health issues	186	49.7	178	58.7	5.11 (0.09)	0.024
Major life stressors for parent(s)/primary caregiver(s) in last 90 days	133	35.6	114	37.6	0.23 (0.02)	0.636
Conflict with or repeated criticism of family	63	16.8	69	22.8	3.38 (0.07)	0.066
Unpaid respite supports unavailable	112	29.9	118	38.9	5.65 (0.09)	0.017
Family went on outings for recreation	331	88.5	277	91.4	1.25 (0.05)	0.263
Child internalizing symptoms	147	39.3	110	36.3	0.52 (-0.03)	0.471
Child externalizing symptoms	134	35.8	101	33.3	0.36 (-0.03)	0.550
Child comorbid internalizing and externalizing symptoms	72	19.3	45	14.9	1.97 (-0.06)	0.161

### Parent/Caregiver with Developmental or Mental Health Issues

More parents/caregivers of children with complex needs were reported to have their own developmental or mental health issues during the pandemic than prior to it [χ^2^ (1, *n* = 745) = 6.49, *p* <.05, *phi* = 0.10], and no significant difference in rates of parental mental health was found between the pandemic period and the post-restrictions period [χ^2^ (1, *n* = 674) = 0.00, *p* =.95, *phi* = − 0.01]. Significantly more parents/caregivers reported having parental mental health/developmental issues in the post-restrictions period compared to the pre-pandemic period [χ^2^ (1, *n* = 677) = 5.11, *p* <.05, *phi* = 0.09].

### Major Life Stressors for Parent(s)/Caregiver(s)

Life stressors were common in all samples, with more parents/caregivers who completed an interRAI Child/Youth assessment during the COVID-19 pandemic indicating major life stressors in the last 90 days compared to parents/caregivers who completed the assessment before the pandemic [χ^2^ (1, *n* = 745) = 22.51, *p* <.001, *phi* = 0.18], and after the pandemic [χ^2^ (1, *n* = 674) = 15.46, *p* <.001, *phi* = − 0.15], and comparable rates between the post-restrictions and pre-pandemic periods [χ^2^ (1, *n* = 677) = 0.23, *p* =.64, *phi* = 0.02] were found.

### Child/Youth Conflict within Family

The rate of family conflict with the child/youth seeking services was higher during the pandemic compared to the pre-pandemic period [χ^2^ (1, *n* = 745) = 5.03, *p* <.05, *phi* = 0.09]. No significant difference was found between the pandemic and post-restrictions periods [χ^2^ (1, *n* = 674) = 0.04, *p* =.84, *phi* = − 0.01]. More families reported conflict with the child/youth during the post-restrictions period than during the pre-pandemic period, but this difference was not significant [χ^2^ (1, *n* = 677) = 3.38, *p* =.07, *phi* = 0.07].

### Unpaid Respite Supports

Unpaid respite support was highest pre-pandemic. Less support during the pandemic was reported but not statistically significant when compared to the pre-pandemic period [χ^2^ (1, *n* = 745) = 3.20, *p* =.07, *phi* = 0.07]. The rate of families reporting that respite was unavailable was comparable between the pandemic and post-restrictions periods [χ^2^ (1, *n* = 674) = 0.36, *p* =.55, *phi* = 0.03]. Rates of families reporting unavailable respite was significantly higher during the post-restrictions period as compared to the pre-pandemic period [χ^2^ (1, *n* = 677) = 5.65, *p* <.05, *phi* = 0.09].

### Family Outings

More families reported going on recreational outings before the pandemic than during the pandemic [χ^2^ (1, *n* = 745) = 20.57, *p* <.001, *phi* = − 0.17]. After restrictions were lifted, the rates of families reporting going on family outings was higher compared to during the pandemic [χ^2^ (1, *n* = 674) = 28.47, *p* <.001, *phi* = 0.21], and comparable to rates from before the pandemic [χ^2^ (1, *n* = 677) = 1.25, *p* =.26, *phi* = 0.05].

### Child/Youth Symptomology

There were no significant differences in internalizing symptoms between the pre-pandemic and pandemic samples [χ^2^ (1, *n* = 745) = 0.95, *p* =.33, *phi* = − 0.04], pandemic and post-restrictions samples [χ^2^ (1, *n* = 674) = 0.01, *p* =.91, *phi* = 0.01], and between the pre-pandemic and post-restrictions samples [χ^2^ (1, *n* = 677) = 0.52, *p* =.47, *phi* = − 0.03].

There were no significant differences in externalizing symptoms between the pre-pandemic and pandemic samples [χ^2^ (1, *n* = 745) = 0.29, *p* =.59, *phi* = − 0.02], pandemic and post-restrictions samples [χ^2^ (1, *n* = 674) = 0.00, *p* =.99, *phi* = 0.00], and between the pre-pandemic and post-restrictions samples [χ^2^ (1, *n* = 677) = 0.36, *p* =.55, *phi* = − 0.03].

More children/youth had higher comorbidity of internalizing and externalizing symptoms in the pre-pandemic sample than in the pandemic sample [χ^2^ (1, *n* = 745) = 8.44, *p* <.01, *phi* = − 0.11]. Samples did not differ in rates of comorbidity between the pandemic and the post-restrictions periods [χ^2^ (1, *n* = 674) = 1.55, *p* =.21, *phi* = 0.05] and between pre-pandemic and post-restriction samples [χ^2^ (1, *n* = 677) = 1.97, *p* =.16, *phi* = − 0.06].

## Discussion

In the present cross-sectional study, comparable pre-pandemic, pandemic, and post-restrictions clinical intake samples were assessed by trained intake professionals who used the same psychometrically acceptable instruments at one large children’s agency. This study investigated Canadian families with children or youth who sought psychiatric assessment, mental health and/or developmental services for complex challenges and special needs for which they had previously received attention. While our archival database on over 1000 children analyzed no direct questions about COVID-19 coping, stress or trauma, we can infer how massive societal changes impacted families with children with special needs by comparing intake clinical data from different timepoints over a four year timespan. As predicted, when compared to a pre-COVID sample, more parents who sought mental health services for their children during public health restrictions in 2020 and 2021 reported higher concerns with their own mental health, life stressors, conflict at home, and less community engagement. Acute stress levels increased in parents of clinic referents during the pandemic lockdowns (36% pre-pandemic, 53% during the pandemic, 38% during post-restrictions); we did not measure the types of stress experienced. Many parents had adult developmental or mental health needs of their own at clinic intake (50% pre-pandemic, 59% during the pandemic, 59% during post-restrictions). When restrictions were lifted in 2022–2023 the majority of caregivers of children with complex needs reported struggling with their mental health, at rates similar to those who sought services during the pandemic. We are adding to the extant literature by reporting results that suggest that most parents of children with complex needs struggled with stress, family conflicts, and their mental health during the COVID-19 pandemic and may still be psychologically vulnerable even after life appears to be back to “normal.”

Given the rudimentary nature of our dependent measures, replication of these findings is needed. We did not find other published child/youth clinic data reported across the 2019 to 2024 timeframe, and we anticipate other children’s mental health clinics will soon report basic descriptive statistics of trends over the past four years. Ideally, large existing research databases of children with special needs could repeat and update (post 2022) measures of parent, child and family well-being. Given the lack of post-COVID comparative analyses in the literature, exploration of ongoing mental health concerns in caregivers of children with special needs is warranted by both clinicians and researchers.

Our study found internalizing and externalizing symptom scores were uniformly high but did not differ across clinical samples, and rates for children’s comorbidity symptoms were higher *before* the pandemic. These cross-sectional samples were collected from tertiary children’s service agency records, representing families with children who have complex needs, high comorbid symptoms and family dysfunction. To qualify for services at this agency, families are required to have received and exhausted previous services elsewhere. In other words, agency intake criteria result in symptoms necessarily falling into the high clinical range (typically 2% of the population). Cross-sample consistency may therefore reflect restriction-of-range and ceiling effects in our data. The finding that there were higher rates of children/youth with more extensive comorbid internalizing/externalizing symptom scores before the pandemic could represent a protective impact of the pandemic for children with clinical needs because these children likely left their home less frequently, therefore expectations and social opportunities likely decreased for these children/youth during the pandemic lockdowns. Children and youth with high levels of anxiety or impulsivity may enjoy not being forced into social situations, and not getting into trouble at schools that were closed.

The number of families seeking child clinical services who continued with recreational outings during the pandemic restrictions was surprising to our team (sample 1 88%, sample 2 75%, sample 3 91%). As predicted the reduction in outings was statistically significant, similar to population survey data (e.g., Moore et al., 2020; Koga et al., 2023). Because children and youth with complex needs may be at risk for under-involvement in formal team/club activities, it may be beneficial to explore the nature of post-pandemic family engagement in recreational activities and whether this directly aided resilience and coping in children. The database does not tell us how families switched from formal to informal activities when public health guidelines mandated social distancing and the closure of recreation venues and school gyms (Ontario Newsroom, 2020; Public Health Ontario, 2020). An Ontario population survey indicates 60 minutes of physical activity levels per day in children pre-COVID was reported by 63%, during lockdown by 21%, and post-lockdown by 54% (Szpunar et al., 2023). Family members’ physical activity impacts mental health (Korczak et al., 2017; Salmon, 2001), and getting adequate exercise as a family was correlated with better child and parent coping during the early stages of the pandemic lockdown (Gadermann et al., 2021; Giannotti et al., 2022; Koga et al., 2023; Thomson et al., 2021; Tso et al., 2020). More time spent together [e.g., shared meals, nature walks (Offord Centre for Child Studies, 2020)] promoted closeness during quarantine (Patrick et al., 2020), and a child’s sense of connectedness to caregivers was negatively related to child mental health symptoms and positively related to happiness during COVID-19 (McArthur et al., 2023). Further, spending quality time with caregivers during the pandemic has been shown to have a positive effect on children’s social and emotional development (Hanley et al., 2024). We therefore recommend new research surveys ask specifically about all types of recreational activities post-COVID: new and old, individual and family, structured and unstructured. We encourage clinicians interested in assessing resilience in families with children with special needs to monitor the quantity and quality of positive family activities as part of basic clinical practice.

The need for more unpaid respite supports was highest in the post-pandemic clinical sample, with 39% reporting this as unavailable (vs. 30% pre-COVID). Curiously (and somewhat similar to Adams et al. reporting on their cerebral palsy sample in 2024), with restrictions lifted, why did rates of unpaid supports not return to pre-pandemic levels? During the pandemic, Wang and Zhang et al. (2022) reported a link between worsening relationships with informal supports and increased risk for poor resilience. Coupled with findings that our caregivers are still in need of informal support after the pandemic, we speculate that caregivers are coming out of the pandemic with lower resilience and may therefore still be too overwhelmed to reach out to those with whom they have lost touch during the lockdowns. Another possible explanation could be that parental mental health challenges may be preventing them from asking for support. Studies have shown that those with mental health needs struggle to ask for respite in part due to stigma, wanting to be self-reliant, and the potential impact that receiving support might have on their relationships (Griffiths et al., 2011; Gulliver et al., 2010). Our data indicates many parents of children with special needs had their own mental health needs. Given this is clinical tertiary intake data, it is positive to report that a majority of referents indicated informal support was available to them across all three cohorts. We do not know the nature of the supports. Child clinicians should routinely ask their clients about emotional and instrumental supports each family member utilizes, and about any losses in informal support since the COVID-19 lockdowns.

The pre-COVID timeframe was associated with slightly lower rates of family conflict at clinic intake (17% of referents pre-pandemic, 24% during, and 23% in the post-restrictions sample). We anticipated the inability to take a break from family would have impacted family arguments to a greater degree in our lockdown sample of families with parents and children reporting significant mental health concerns (e.g., Wolstencroft et al., 2021). The low base rates of reported conflict seen presently may be an artifact of the single item coded (family conflict or criticism), or of guardian concerns about self-reporting family dysfunction to a clinic intake worker. In our study this is partially mitigated by intake workers trained on the ChYMH and ChYMH-DD to utilize all information (not just parent report), including previous reports to determine item ratings. More in-depth and valid assessment of family functioning during and after pandemic stressors is advised, with careful consideration of covariates that are distal stressors not available in the present dataset (employment, income, health and education changes).

In the population research reviewed, most people facing societal changes due to COVID-19 continued to cope. Comprehensive models of risk and resiliency in family functioning during the pandemic were proposed early in the COVID-19 crisis to aid research direction (Prime et al., 2020). Child psychopathology researchers note that cumulative stressor effects can lead to a developmental cascade across cognitive, behavioural, and emotional domains and within family systems in a nature-nurture transactional fashion, helping to explain the comorbidities and correlated outcomes seen across family members in the literature (see Masten & Cicchetti, 2010; Prime et al., 2020). These theorists note that family adjustment is influenced by interactive distal (e.g., economic losses) and proximal (altered interpersonal contacts) changes that likely have greater impact on families with pre-existing vulnerabilities. Parents/caregivers seeking mental health services for children commonly describe high rates of pre-existing adversity including family dysfunction and both child-related and personal stress (Gassman-Pines et al., 2020; Meraj et al., 2023; Noh et al., 1989). Three years after the pandemic began, our clinic base rate data on those seeking more intensive services for their child or youth with high needs indicates the majority of parents reported personal mental health challenges (59%) and a notable proportion indicated needing more respite (39%). Kaltenbach et al. (2023) reported a relation between COVID-19 stressors (e.g., inability for child to receive necessary treatment, increase in emotional/financial stresses) and symptoms of post-traumatic stress disorder (PTSD) in parents of children with intellectual and neurodevelopmental disorders, with more than half their sample meeting the criteria for a PTSD diagnosis. Wang and Zhang et al. (2022) found that parents of children with autism were less resilient and had more symptoms of anxiety and depression than parents of typically developing children, with improvements in mental well-being not demonstrated when restrictions were loosened. We contend that our clinical samples of children with high needs and parents who had pre-existing mental health concerns represent a group with unique vulnerabilities deserving of increased support. Post-COVID research is required to understand the frequency or magnitude of possible real world functional outcomes on special needs families, such as family dissolution and school failure rates.

Our data strongly indicate that child and parent mental health services need an integrated focus, for example child mental health clinicians must find a means to routinely assess caregiver well-being, likely best initiated by using a broad formal intake screener such as used in this study. Professionals can also quickly and systematically screen for potential impacts of the COVID-19 pandemic by considering both new and historical adverse experiences across family members. Further, our data clearly indicate that child mental health agencies require a family support focus in their clinician hiring and programming, and the pandemic heightened the urgency of this service recommendation. For a thorough discussion and recommendations on child mental health organization and post COVID services system changes to consider in simultaneously supporting multiple family members who report they are not coping, see Sanders et al. (2024). The COVID-19 pandemic also reminds us of the need for trauma-informed practices and policies (Collin-Vézina et al., 2020). Sharing the news with your clients that other parents have reported sad/anxious feelings both during and after the pandemic, will hopefully foster a sense of community and promote behaviours and supports that strengthen resilience. By asking “What happened to you and your family?” we may arrive at a greater understanding of what families have undergone before and after the pandemic (Perry & Winfrey, 2021), aiding rapport and treatment goal setting (Fisher et al., 2020).

From a researcher perspective, several observations stand out from this review of the empirical literature. It is recommended that as cross-sectional and longitudinal data are published from the pre-COVID, during COVID, and post-COVID restrictions years, these “pre, post” search terms be clearly listed as key words to aid rapid literature searching. Second, while the initial date of March 2020 is fairly common as the start of COVID restrictions and marks the beginning of the “during” subsample, dates at which COVID lockdowns eased differ from country to country and within countries. It is essential that when using the term “post” COVID or “post” restrictions/lockdowns, dates linked to some definitional marker be described. For example, presently our post-cohort sample begins in the summer of 2022 and is marked by schools remaining fully open throughout the 2022–2023 school year. Our data report on sample differences on a rudimentary scale, and this is true of much of the literature reviewed. Linking this scale data to objective markers of functional changes for individuals and families (e.g., school achievement, marital separation, hospitalization) is recommended. Finally, some authors focus discussion solely on the exacerbation of symptoms or conflict during COVID that was found in a minority of their overall population surveys, while failing to also note that a significant majority of families, in the population research reviewed here, report they are not in crisis. Greater attention to resilience factors is warranted in both clinical and non-clinical samples.

### Limitations

The items in the archival dataset used for the analyses in this study were established prior to the pandemic. The clinical intake assessment does not contain any items specific to COVID-19. Key variables of interest were not found in the clinical database, for example no adequate measure of financial stress/job loss, COVID illness, loss experiences in family members, or other pandemic-specific stressors were available (e.g., McArthur et al., 2023; Prime et al., 2022). We did not have data on self-perceived pandemic benefits. The limited items and simple binary (Yes/No) scaling limited analyses.

The data report on families before, during, and after pandemic restrictions were lifted at one children’s tertiary service agency. Data from other agencies would have aided generalizability of the results. We did not include families who were receiving services during the initial months of the pandemic. At the start of the pandemic this agency made the decision to switch to the shorter interRAI ChYMH-Screener temporarily (six months) to reduce intake burden on families, the ChYMH-Screener has fewer items than the interRAI ChYMH and ChYMH-DD tools, therefore it does not have all the variables that were of interest for this study. Hence, we did not include a timepoint for the earliest period of the COVID-19 pandemic. Having a group during the initial months of the pandemic would have helped us to examine the acute vs. longer term adjustment to pandemic changes for families with a clinical referral to this agency. Our pandemic sample represents families who had already been experiencing life uncertainty and restriction drain for a few months. Finally, it was not our goal to compare the experiences of this clinical sample to experiences of non-clinical samples.

Our three samples were mean age 11, however a higher proportion of adolescents were in the post-restrictions sample as compared to the pre-pandemic sample. It is possible that the sample ages may have played an impact on post-pandemic recovery, although we expect that families of younger children are likely experiencing more long-term impacts due to the extra care these children required (Marchetti et al., 2020).

The current design is cross-sectional in nature and we can not know if cohort differences relate to COVID-19. A repeated measures design over multiple years would have been ideal following the same individuals, but not possible in a clinic intake archival study. Although we do have a post-restrictions group, longer term pandemic impacts on the family require further time samples in 2025 and beyond.

## Conclusion

In a cross-sectional study of tertiary clinical intake data at a children’s service agency, significant mental health and respite concerns were evident in all samples of children and youth and their parents over the four years included in the study data. As expected, more parents who sought services for their children during acute social lockdowns (2020–2021) reported concerns with their own mental health, major life stressors, conflict at home, and less community engagement than a similar pre-COVID (2019-early 2020) sample. We offer a first look at family functioning in this high-needs population in the post-COVID lockdown timeframe (2022–2023). This cross-sectional study indicates that those caring for children with significant needs may still be experiencing mental health challenges and be in need of respite in the post-lockdown era, despite reports of acute stressors in 2023 returning to pre-COVID levels. Optimistically, very high reports of recreational outings after the restrictions were lifted indicate that post-COVID coping strategies are being used by most families referred to the tertiary agency. Clinicians working with children with complex combinations of needs and their families may benefit from attention to all family members and a trauma-informed care approach to understand the impacts of the COVID-19 pandemic response in the context of historical adversity for that family.

## Data Availability

The data used in this study consist of client information and, due to privacy regulations under the Personal Health Information Protection Act (PHIPA), cannot be shared and may only be disclosed in compliance with PHIPA.

## References

[CR1] Achterberg, M., Dobbelaar, S., Boer, O. D., & Crone, E. A. (2021). Perceived stress as mediator for longitudinal effects of the COVID-19 lockdown on wellbeing of parents and children. *Scientific Reports*, *11*, 2971. 10.1038/s41598-021-81720-8 33536464 10.1038/s41598-021-81720-8 PMC7859207

[CR2] Adams, S., Moosa, A., & Bhorat, R. (2024). A socioecological framing of the experiences of caregivers of children with cerebral palsy in South Africa post COVID-19. *Journal of Child Neurology*. 10.1177/08830738241292844 39587934 10.1177/08830738241292844 PMC11909768

[CR3] American Psychological Association (2020a, May). *Stress in America™ 2020: Stress in the time of COVID-19 (volume one)*. https://www.apa.org/news/press/releases/stress/2020/stress-in-america-covid.pdf

[CR4] American Psychological Association (2020b, June). *Stress in America™ 2020: Stress in the time of COVID-19 (volume two).* https://www.apa.org/news/press/releases/stress/2020/stress-in-america-covid-june.pdf

[CR5] Arim, R., Findlay, L., & Kohen, D. (2020, August 27). The impact of the COVID-19 pandemic on Canadian families of children with disabilities. *StatCan COVID-19: Data to Insights for a Better Canada, Catalogue no. 45280001* https://www150.statcan.gc.ca/n1/en/pub/45-28-0001/2020001/article/00066-eng.pdf?st=Z3C4YtCJ

[CR6] Badr, H. S., Du, H., Marshall, M., Dong, E., Squire, M. M., & Gardner, L. M. (2020). Association between mobility patterns and COVID-19 transmission in the USA: A mathematical modelling study. *Lancet Infectious Diseases*, *20*, 1247–1254. 10.1016/S1473-3099(20)30553-3 32621869 10.1016/S1473-3099(20)30553-3 PMC7329287

[CR7] Baker, B. L., McIntyre, L. L., Blacher, J., Crnic, K., Edelbrock, C., & Low, C. (2003). Pre-school children with and without developmental delay: Behaviour problems and parenting stress over time. *Journal of Intellectual Disability Research*, *47*(4–5), 217–230. 10.1046/j.1365-2788.2003.00484.x 12787154 10.1046/j.1365-2788.2003.00484.x

[CR8] Benke, C., Autenrieth, L. K., Asselmann, E., & Pané–Farré, C. A. (2023). One year after the COVID-19 outbreak in Germany: Long-term changes in depression, anxiety, loneliness, distress and life satisfaction. *European Archives of Psychiatry and Clinical Neuroscience*, *273*, 289–299. 10.1007/s00406-022-01400-0 35348855 10.1007/s00406-022-01400-0 PMC8962282

[CR9] Bentenuto, A., Mazzoni, N., Giannotti, M., Venuti, P., & de Falco, S. (2021). Psychological impact of COVID-19 pandemic in Italian families of children with neurodevelopmental disorders. *Research in Developmental Disabilities, 109*. 10.1016/j.ridd.2020.103840 10.1016/j.ridd.2020.103840 PMC9186313 33383468

[CR10] Biggs, E. E., Zhang, L. S., & Ling, C. (2024). Stress and resilience of families with children with intellectual and developmental disabilities during the COVID-19 pandemic: A mixed-methods study. *Research and Practice for Persons with Severe Disabilities*, *49*(4), 242–261. 10.1177/15407969241263519

[CR11] Bronfenbrenner, U. (1979). *The ecology of human development*. Harvard University Press.

[CR12] Brown, S. M., Doom, J. R., Lechuga-Pena, S., Watamura, S. E., & Koppels, T. (2020). Stress and parenting during the global COVID-19 pandemic. *Child Abuse & Neglect*, *110*(2). 10.1016/j.chiabu.2020.104699 10.1016/j.chiabu.2020.104699 PMC7440155 32859394

[CR13] Camargo, D., Navarro-Tapia, E., Pérez-Tur, J., & Cardona, F. (2023). Relationship between COVID-19 pandemic confinement and worsening or onset of depressive disorders. *Brain Sciences*, *13*(6), 899. 10.3390/brainsci13060899 37371377 10.3390/brainsci13060899 PMC10296361

[CR14] Campione-Barr, N., Skinner, A., Moeller, K., Cui, L., Kealy, C., & Cookston, J. (2024). The role of family relationships on adolescents’ development and adjustment during the COVID-19 pandemic: A systematic review. *Journal of Research on Adolescence*. 10.1111/jora.12969 38727698 10.1111/jora.12969

[CR15] Cimino, S., Di Vito, P., & Cerniglia, L. (2024). The impact of COVID-19 pandemic on psychopathological symptoms in mothers and their school-age children before, during and after the COVID-19 pandemic peak. *Current Psychology*, *43*(14), 13100–13109. 10.1007/s12144-022-03360-z 10.1007/s12144-022-03360-z PMC9243995 35789629

[CR16] Cluver, L., Lachman, J. M., Sherr, L., Wessels, I., Krug, E., Rakotomalala, S., Blight, S., Hillis, S., Bachman, G., Green, O., Butchart, A., Tomlinson, M., Ward, C. L., Doubt, J., & McDonald, K. (2020). Parenting in a time of COVID-19. *The Lancet, 395*, e64. 10.1016/S0140-6736(20)30736-4 10.1016/S0140-6736(20)30736-4 PMC7146667 32220657

[CR18] Colizzi, M., Sironi, E., Antonini, F., Ciceri, M. L., Bovo, C., & Zoccante, L. (2020). Psychosocial and behavioral impact of COVID-19 in autism spectrum disorder: An online parent survey. *Brain Sciences*, *10*, 341. 10.3390/brainsci10060341 32503172 10.3390/brainsci10060341 PMC7349059

[CR19] Coller, R. J., DeMuri, G. P., Eickhoff, J. C., Singh-Verdeflor, K., Warner, G., Butteris, S. M., Ehlenbach, M. L., Gerber, D., Katz, B., Koval, S., & Kelly, M. M. (2024). School perceptions and attendance for children with medical complexity during COVID‐19 over time. *The Journal of School Health*, *94*(11), 1009–1018. 10.1111/josh.13503 39228276 10.1111/josh.13503 PMC11521758

[CR20] Collin-Vézina, D., Brend, D., & Beeman, I. (2020). When it counts the most: Trauma-informed care and the COVID-19 global pandemic. *Developmental Child Welfare*, *2*(3), 172–179. 10.1177/2516103220942530

[CR21] Elliott, S. (2022, January 21). *Ontario’s COVID-19 response: A history of announced measures, 2020–2022*. JD Supra. https://www.jdsupra.com/legalnews/ontario-s-covid-19-response-a-history-1280608/

[CR22] Essler, S., Christner, N., & Paulus, M. (2024). Short-term and long-term effects of the COVID-19 pandemic on child psychological well-being: A four-wave longitudinal study. *European Child & Adolescent Psychiatry*, *33*, 909–922. 10.1007/s00787-023-02215-7 37119393 10.1007/s00787-023-02215-7 PMC10148581

[CR23] Fadiya, B. (2019, October). *Validation of ChYMH scales and cut-points for ChYMH-DD use. Presentation to staff*. Child and Parent Resource Institute.

[CR24] Fadiya, B. (2016, November). *Determining interRAI scale cut-points using receiver operating characteristic (ROC) curves. Presentation to staff*. Child and Parent Resource Institute.

[CR25] Fancourt, D., Steptoe, A., & Bu, F. (2021). Trajectories of anxiety and depressive symptoms during enforced isolation due to COVID-19 in England: A longitudinal study. *The Lancet Psychiatry*, *8*(2), 141–149. 10.1016/S2215-0366(20)30482-X 33308420 10.1016/S2215-0366(20)30482-X PMC7820109

[CR26] Feinberg, M. E., Mogle, J. A., Lee, J. K., Tornello, S. L., Hostetler, M. L., Cifelli, J. A., Bai, S., & Hotez, E. (2022). Impact of the COVID-19 pandemic on parent, child, and family functioning. *Family Process*, *61*(1), 361–374. 10.1111/famp.12649 33830510 10.1111/famp.12649 PMC8250962

[CR27] Findlay, L., & Arim, R. (2020, April 24). Canadians report lower self-perceived mental health during the COVID-19 pandemic. *StatCan COVID-19: Data to insights for a better Canada, Catalogue no. 45280001*. https://www150.statcan.gc.ca/n1/pub/45-28-0001/2020001/article/00003-eng.htm?fbclid=IwAR3hqELORNXB9D9FZHIAVeaUk7fVmujyeXw7RvDMk5g5BsyvU_ZNhncz65A

[CR28] Fisher, E. B., Miller, S. M., Evans, M., Luu, S. L., Tang, P. Y., Valovcin, D. D., & Castellano, C. (2020). COVID-19, stress, trauma, and peer support – observations from the field. *Translational Behavioral Medicine*, *10*, 503–505. 10.1093/tbm/ibaa056 32569372 10.1093/tbm/ibaa056 PMC7337751

[CR29] Freeman, J. (2023, March 17). *Ontario declared a state of emergency over the COVID-19 pandemic 3 years ago today. Here’s a look back.* https://toronto.ctvnews.ca/ontario-declared-a-state-of-emergency-over-the-covid-19-pandemic-3-years-ago-today-here-s-a-look-back-1.6317337

[CR30] Gadermann, A. C., Thomson, K. C., Richardson, C. G., Gagné, M., McAuliffe, C., Hirani, S., & Jenkins, E. (2021). Examining the impacts of the COVID-19 pandemic on family mental health in Canada: Findings from a national cross-sectional study. *British Medical Journal Open*, *11*(1), e042871. 10.1136/bmjopen-2020-042871 10.1136/bmjopen-2020-042871 PMC7804831 33436472

[CR31] Gassman-Pines, A., Ananat, E. O., & Fitz-Henley, J. (2020). COVID-19 and parent-child psychological well-being. *Pediatrics*, *146*(4), e2020007294. 10.1542/peds.2020-007294 32764151 10.1542/peds.2020-007294 PMC7546085

[CR32] Gayatri, M., & Irawaty, D. K. (2022). Family resilience during COVID-19 pandemic: A literature review. *The Family Journal: Counseling and Therapy for Couples and Families*, *30*(2), 132–138. 10.1177/10664807211023875 10.1177/10664807211023875 PMC8980846 35399750

[CR33] Geprägs, A., Bürgin, D., Fegert, J. M., Brahler, E., & Clemens, V. (2024). Trends in changes of family functioning during different phases of the pandemic – findings across four population-based surveys between 2020 to 2023 in Germany. *BMC Public Health*, *24*, 3230. 10.1186/s12889-024-20650-2 39567945 10.1186/s12889-024-20650-2 PMC11580525

[CR34] Giannotti, M., Mazzoni, N., Bentenuto, A., Venuti, P., & de Falco, S. (2022). Family adjustment to COVID-19 lockdown in Italy: Parental stress, coparenting, and child externalizing behavior. *Family Process*, *61*(2), 745–763. 10.1111/famp.12686 34195986 10.1111/famp.12686 PMC8444949

[CR35] Goldberg, A. E., Allen, K. R., & Smith, J. Z. (2021). Divorced and separated parents during the COVID-19 pandemic. *Family Process*, *60*, 866–887. 10.1111/famp.12693 34227099 10.1111/famp.12693 PMC8444689

[CR36] Griffiths, K. M., Crisp, D. A., Barney, L., & Reid, R. (2011). Seeking help for depression from family and friends: A qualitative analysis of perceived advantages and disadvantages. *BMC Psychiatry*, *11*, 196. 10.1186/1471-244X-11-196 22171567 10.1186/1471-244X-11-196 PMC3271042

[CR37] Gulliver, A., Griffiths, K. M., & Christensen, H. (2010). Perceived barriers and facilitators to mental health help-seeking in young people: A systematic review. *BMC Psychiatry*, *10*, 113. 10.1186/1471-244X-10-113 21192795 10.1186/1471-244X-10-113 PMC3022639

[CR38] Hanley, A., Symonds, J. E., & Horan, J. (2024). COVID-19 school closures and children’s social and emotional functioning: The protective influence of parent, sibling, and peer relationships. *Education 3–13*, *52*(8), 1452–1463. 10.1080/03004279.2022.2154615

[CR39] Harris, M. N., & Konkel, R. H. (2024). Safer or endangered at home? An examination of neighborhood effects on family violence before, during, and after the COVID-19 safer-at-home order. *American Journal of Criminal Justice*, *49*(6), 842–866. 10.1007/s12103-024-09772-w

[CR40] Hawke, L. D., Barbic, S. P., Voineskos, A., Szatmari, P., Cleverley, K., Hayes, E., Relihan, J., Daley, M., Courtney, D., Cheung, A., Darnay, K., & Henderson, J. (2020). Impacts of COVID-19 on youth mental health, substance use, and well-being: A rapid survey of clinical and community samples. *The Canadian Journal of Psychiatry/ La Revue Canadienne De Psychiatrie*, *65*(10), 701–709. 10.1177/0706743720940562 10.1177/0706743720940562 PMC7502874 32662303

[CR41] Hayes, S. A., & Watson, S. L. (2013). The impact of parenting stress: A meta-analysis of studies comparing the experience of parenting stress in parents of children with and without autism spectrum disorder. *Journal of Autism and Developmental Disorders*, *43*, 629–642. 10.1007/s10803-012-1604-y 10.1007/s10803-012-1604-y 22790429

[CR42] Hill, A. P., Zuckerman, K. E., Hagen, A. D., Kriz, D. J., Duvall, S. W., van Santen, J., Nigg, J., Fair, D., & Fombonne, E. (2014). Aggressive behavior problems in children with autism spectrum disorders: Prevalence and correlates in a large clinical sample. *Research in Autism Spectrum Disorders*, *8*(9), 1121–1133. 10.1016/j.rasd.2014.05.006 25221619 10.1016/j.rasd.2014.05.006 PMC4160737

[CR43] Hirdes, J. P., Ljunggren, G., Morris, J. N., Frijters, D. H. M., Soveri, H. F., Gray, L., Björkgren, M., & Gilgen, R. (2008). Reliability of the interRAI suite of assessment instruments: A 12-country study of an integrated health information system. *BMC Health Services Research*, *8*, 277. 10.1186/1472-6963-8-277 19115991 10.1186/1472-6963-8-277 PMC2631461

[CR44] Isensee, C., Schmid, B., Marschik, P. B., Zhang, D., & Poustka, L. (2022). Impact of COVID-19 pandemic on families living with autism: An online survey. *Research in Developmental Disabilities*, *129*, 104307. 10.1016/j.ridd.2022.104307 35908370 10.1016/j.ridd.2022.104307 PMC9271458

[CR45] Jacques, C., Saulnier, G., Éthier, A., & Soulières, I. (2022). Experience of autistic children and their families during the pandemic: From distress to coping strategies. *Journal of Autism and Developmental Disorders*, *52*, 3626–3638. 10.1007/s10803-021-05233-z 34448994 10.1007/s10803-021-05233-z PMC8391854

[CR46] Janus, M., Mauti, E., Horner, M., Duku, E., Siddiqua, A., & Davies, S. (2018). Behavior profiles of children with autism spectrum disorder in kindergarten: Comparison with other developmental disabilities and typically developing children. *Autism Research*, *11*(3), 410–420. 10.1002/aur.1904 29219245 10.1002/aur.1904

[CR47] Kaltenbach, E., Xiong, T., Thomson, D., & McGrath, P. J. (2023). Challenges of parents with children with intellectual and neurodevelopmental disorders during COVID-19: Experiences and their impact on mental health. *Journal of Intellectual & Developmental Disability*, *48*(1), 77–84. 10.3109/13668250.2022.2096955 39815860 10.3109/13668250.2022.2096955

[CR48] Knedlíková, L., Dědková, L., Kolář, S., Česká, K., Vyhnalová, M., Stroupková, L., Pejčochová, J., Pavel, T., Lacko, D., Horák, O., Ošlejšková, H., & Danhofer, P. (2024). The impact of the COVID-19 pandemic on stress and coping in parents of children with Autism Spectrum Disorder. *PloS One*, *19*(11), e0313426. 10.1371/journal.pone.0313426 39509445 10.1371/journal.pone.0313426 PMC11542825

[CR49] Koga, T., Okubo, R., Chen, C., Hagiwara, K., Mizumoto, T., Nakagawa, S., & Tabuchi, T. (2023). Associations of parent-child exercise with family relations and parental mental health during the COVID-19 pandemic. *Journal of Affective Disorders*, *324*, 551–558. 10.1016/j.jad.2023.01.001 36623559 10.1016/j.jad.2023.01.001 PMC9816069

[CR50] Kong, M. (2021). What COVID-19 means for non-neurotypical children and their families. *Pediatric Research*, *89*, 396–397. 10.1038/s41390-020-0913-7 32316029 10.1038/s41390-020-0913-7 PMC7979535

[CR51] Korczak, D. J., Madigan, S., & Colasanto, M. (2017). Children’s physical activity and depression: A meta-analysis. *Pediatrics*, *139*(4), e20162266. 10.1542/peds.2016-2266 28314824 10.1542/peds.2016-2266

[CR52] Larsen, L., Schauber, S. K., Holt, T., & Helland, M. S. (2023). Longitudinal COVID-19 effects on child mental health: Vulnerability and age dependent trajectories. *Child and Adolescent Psychiatry and Mental Health*, *17*, 104. 10.1186/s13034-023-00652-5 37667287 10.1186/s13034-023-00652-5 PMC10476387

[CR53] Latzer, I. T., Leitner, Y., & Karnieli-Miller, O. (2021). Core experiences of parents of children with autism during the COVID-19 pandemic lockdown. *Autism*, *25*(4), 1047–1059. 10.1177/1362361320984317 33435701 10.1177/1362361320984317

[CR55] Lau, C., Stewart, S. L., Saklofske, D. H., & Hirdes, J. (2019). Scale development and psychometric properties of internalizing symptoms: The interRAI Child and Youth Mental Health internalizing subscale. *Psychiatry Research*, *278*, 235–241. 10.1016/j.psychres.2019.06.013 31228857 10.1016/j.psychres.2019.06.013

[CR54] Lau, C., Stewart, S. L., Saklofske, D. H., & Hirdes, J. (2021). Development and psychometric validation of the interRAI ChYMH externalizing subscale. *Clinical Child Psychology and Psychiatry*, *26*(1), 295–305. 10.1177/1359104520963143 33066721 10.1177/1359104520963143

[CR56] Lee, J. (2020). Mental health effects of school closures during COVID-19. *The Lancet Child & Adolescent Health*, *4*(6), 421. 10.1016/S2352-4642(20)30109-7 32302537 10.1016/S2352-4642(20)30109-7 PMC7156240

[CR57] Lee, S. J., Ward, K. P., Chang, O. D., & Downing, K. M. (2021). Parenting activities and the transition to home-based education during the COVID-19 pandemic. *Children and Youth Services Review*, *122*. 10.1016/j.childyouth.2020.105585 10.1016/j.childyouth.2020.105585 PMC7553006 33071407

[CR58] Lipkin, M., & Crepeau-Hobson, F. (2023). The impact of the COVID-19 school closures on families with children with disabilities: A qualitative analysis. *Psychology in the Schools*, *60*(5), 1544–1559. 10.1002/pits.22706 10.1002/pits.22706 PMC9088372 35572177

[CR59] MacDonald, H., & Callery, P. (2004). Different meanings of respite: A study of parents, nurses and social workers caring for children with complex needs. *Child: Care Health & Development*, *30*(3), 279–288. 15104588 10.1111/j.1365-2214.2004.00392.x

[CR60] Manis, J., & Stewart, S. L. (2024). A snapshot of peer relationships in children and youth: Pre- versus during COVID-19. *International Journal of Environmental Research and Public Health*, *21*, 1552. 10.3390/ijerph21121552 39767395 10.3390/ijerph21121552 PMC11675896

[CR61] Marchetti, D., Fontanesi, L., Mazza, C., Di Giandomenico, S., Roma, P., & Verrocchio, M. C. (2020). Parenting-related exhaustion during the Italian COVID-19 lockdown. *Journal of Pediatric Psychology*, *45*(10), 1114–1123. 10.1093/jpepsy/jsaa093 33068403 10.1093/jpepsy/jsaa093 PMC7665691

[CR62] Masten, A., & Cicchetti, D. (2010). Developmental cascades. *Development and Psychopathology*, *22*, 491–495. 10.1017/S0954579410000222 20576173 10.1017/S0954579410000222

[CR64] McArthur, B. A., Racine, N., McDonald, S., Tough, S., & Madigan, S. (2023). Child and family factors associated with child mental health and well-being during COVID-19. *European Child & Adolescent Psychiatry*, *32*, 223–233. 10.1007/s00787-021-01849-9 34302530 10.1007/s00787-021-01849-9 PMC8302979

[CR65] McCann, D., Bull, R., & Winzenberg, T. (2012). The daily patterns of time use for parents of children with complex needs: A systematic review. *Journal of Child Health Care*, *16*(1), 26–52. 10.1177/1367493511420186 22308543 10.1177/1367493511420186

[CR63] Meraj, N., Arbeau, K., Fadiya, B., Ketelaars, T., St. Pierre, J., Swart, G. T., & Zayed, R. (2023). Introducing the Adverse Life Events Inventory for Children (ALEIC): An examination of adverse experiences and related impacts in a large clinical sample of children and youth. *Traumatology*, *29*(2), 137–148. 10.1037/trm0000385

[CR66] Miniarikova, E., Vernhet, C., Peries, M., Loubersac, J., Picot, M. C., Munir, K., & Baghdadli, A. (2022). Anxiety and depression in parents of children with autism spectrum disorder during the first COVID-19 lockdown: Report from the ELENA cohort. *Journal of Psychiatric Research*, *149*, 344–351. 10.1016/j.jpsychires.2021.11.022 34776249 10.1016/j.jpsychires.2021.11.022 PMC9750171

[CR67] Mitchell, R. H., Toulany, A., Chung, H., Cohen, E., Fu, L., Strauss, R., Vigod, S. N., Stukel, T. A., Moran, K., Guttmann, A., Kurdyak, P., Artani, A., Kopec, M., & Saunders, N. R. (2023). Self-harm among youth during the first 28 months of the COVID-19 pandemic in Ontario, Canada: A population-based study. *Canadian Medical Association Journal*, *195*(36), E1210–E1220. 10.1503/cmaj.230127 37722745 10.1503/cmaj.230127 PMC10506509

[CR68] Moore, S. A., Faulkner, G., Rhodes, R. E., Brussoni, M., Chulak-Bozzer, T., Ferguson, L. J., Mitra, R., O’Reilly, N., Spence, J. C., Vanderloo, L. M., & Tremblay, M. S. (2020). Impact of the COVID-19 virus outbreak on movement and play behaviours of Canadian children and youth: A national survey. *International Journal of Behavioral Nutrition and Physical Activity*, *17*. 10.1186/s12966-020-00987-8 10.1186/s12966-020-00987-8 PMC7336091 32631350

[CR69] Nair, M., Moore, K., Sanford, S. J., McNair, A., Matheson, A., & Wong, E. (2024). Impacts of COVID-19 on young children and families: A qualitative study using Best Starts for Kids Health Survey Data in King County, WA.* Maternal and Child Health Journal*, *28*(1), 116–124. 10.1007/s10995-023-03810-5 37922058 10.1007/s10995-023-03810-5 PMC10876728

[CR70] Nielsen, K. (2021, May 10). *A timeline of COVID-19 in Ontario*. Global News. https://globalnews.ca/news/6859636/Ontario-coronavirus-timeline/

[CR71] Noh, S., Dumas, J. E., Wolf, L. C., & Fisman, S. N. (1989). Delineating sources of stress in parents of exceptional children. *Family Relations*, *38*(4), 456–461. 10.2307/585753

[CR72] Offord Centre for Child Studies (2020). *Impact of the COVID-19 pandemic on Ontario families with children: Findings from the initial lockdown.* https://strongfamilies.ca/wp-content/uploads/2020/10/OPS-Executive-Report-EN-FINAL.pdf

[CR73] Ontario Newsroom. (2020, March 30). *News release: Ontario extends emergency declaration to stop the spread of COVID-19*. https://news.ontario.ca/en/release/56523/ontario-extends-emergency-declaration-to-stop-the-spread-of-covid-19

[CR74] Orban, E., Li, L. Y., Gilbert, M., Napp, A. K., Kaman, A., Topf, S., Boecker, M., Devine, J., Reiß, F., Wendel, F., Jung-Sievers, C., Ernst, V. S., Franze, M., Möhler, E., Breitinger, E., Bender, S., & Ravens-Sieberer, U. (2024). Mental health and quality of life in children and adolescents during the COVID-19 pandemic: A systematic review of longitudinal studies. *Frontiers in Public Health*, *11*, 1275917. 10.3389/fpubh.2023.1275917 38259801 10.3389/fpubh.2023.1275917 PMC10800626

[CR75] Painter, F. L., Booth, A. T., Letcher, P., Olsson, C. A., & McIntosh, J. E. (2024). Multilevel impacts of the COVID-19 pandemic: A bioecological systems perspective of parent and child experiences. *Child & Youth Care Forum*, *53*(2), 411–437. 10.1007/s10566-023-09761-w

[CR76] Pallant, J. (2020). *SPSS survival manual: A step by step guide to data analysis using IBM SPSS* (7th ed.). Open University Press.

[CR77] Parsons Leigh, J., Moss, S. J., Sriskandarajah, C., McArthur, E., Ahmed, S. B., Birnie, K., Halperin, D., Halperin, S., Harley, M., Hu, J., Ng Kamstra, J., Leppan, L., Nickel, A., Racine, N., Russell, K., Smith, S., Solis, M., Stelfox, M., Tutelman, P. R., Stelfox, H. T., & Fiest, K. M. (2023). A multi-informant national survey on the impact of COVID-19 on mental health symptoms of parent-child dyads in Canada. *Scientific Reports*, *13*(1), 7972. 10.1038/s41598-023-34544-7 37198202 10.1038/s41598-023-34544-7 PMC10189235

[CR78] Patrick, S. W., Henkhaus, L. E., Zickafoose, J. S., Lovell, K., Halvorson, A., Loch, S., Letterie, M., & Davis, M. M. (2020). Well-being of parents and children during the COVID-19 pandemic: A national survey. *Pediatrics*, *146*(4), e2020016824. 10.1542/peds.2020-016824 32709738 10.1542/peds.2020-016824

[CR79] Perry, B. D., & Winfrey, O. (2021). *What happened to you? Conversations on trauma, resilience, and healing*. Flatiron Books.

[CR80] Pierce, M., Hope, H., Ford, T., Hatch, S., Hotopf, M., John, A., Kontopantelis, E., Webb, R., Wessely, S., McManus, S., & Abel, K. M. (2020). Mental health before and during the COVID-19 pandemic: A longitudinal probability sample survey of the UK population. *The Lancet Psychiatry*, *7*(10), 883–892. 10.1016/S2215-0366(20)30308-4 32707037 10.1016/S2215-0366(20)30308-4 PMC7373389

[CR82] Prematunga, R. K. (2012). Correlational analysis. *Australian Critical Care*, *25*, 195–199. 10.1016/j.aucc.2012.02.003 22464607 10.1016/j.aucc.2012.02.003

[CR84] Prime, H., Wade, M., & Browne, D. T. (2020). Risk and resilience in family well-being during the COVID-19 pandemic. *American Psychologist*, *75*(5), 631–643. 10.1037/amp0000660 32437181 10.1037/amp0000660

[CR83] Prime, H., Wade, M., & Browne, D. T. (2022). Pandemic-related disruption and positive adaptation: Profiles of family function at the onset of the pandemic. *Adversity and Resilience Science*, *3*, 321–333. 10.1007/s42844-022-00077-7 36117857 10.1007/s42844-022-00077-7 PMC9471027

[CR85] Public Health Ontario (2020). *Coronavirus disease 2019 (COVID-19): Physical distancing*. https://www.publichealthontario.ca/-/media/documents/ncov/factsheet/factsheet-covid-19-guide-physical-distancing.pdf?la=en

[CR86] Ravens-Sieberer, U., Devine, J., Napp, A. K., Kaman, A., Saftig, L., Gilbert, M., Reiß, F., Löffler, C., Simon, A. M., Hurrelmann, K., Walper, S., Schlack, R., Hölling, H., Wieler, L. H., & Erhart, M. (2023). Three years into the pandemic: Results of the longitudinal German COPSY study on youth mental health and health-related quality of life. *Frontiers in Public Health*, *11*, 1129073. 10.3389/fpubh.2023.1129073 37397777 10.3389/fpubh.2023.1129073 PMC10307958

[CR87] Ren, J., Li, X., Chen, S., Chen, S., & Nie, Y. (2020). The influence of factors such as parenting stress and social support on the state anxiety in parents of special needs children during the COVID-19 epidemic. *Frontiers in Psychology*, *11*, 565393. 10.3389/fpsyg.2020.565393 33362628 10.3389/fpsyg.2020.565393 PMC7758194

[CR88] Rodman, A. M., Rosen, M. L., Kasparek, S. W., Mayes, M., Lengua, L., Meltzoff, A. N., & McLaughlin, K. A. (2024). Social experiences and youth psychopathology during the COVID-19 pandemic: A longitudinal study. *Development and Psychopathology*, *36*(1), 366–378. 10.1017/S0954579422001250 36503551 10.1017/S0954579422001250 PMC10258229

[CR89] Salmon, P. (2001). Effects of physical exercise on anxiety, depression, and sensitivity to stress: A unifying theory. *Clinical Psychology Review*, *21*(1), 33–61. 11148895 10.1016/s0272-7358(99)00032-x

[CR90] Sanders, J. E., Seale, A., Wilton, L., Carrothers, E., Siddall, M., Pereira, Z., Watson, E., Bernardi, K., Arundel, M. K., & Csiernik, R. (2024). “A better me for my children”: A call for systemic relational family work in child and youth mental health. *Families in Society*. 10.1177/10443894241261743 10.1177/10443894241261743 PMC13046230 41939205

[CR91] Shorey, S., Lau, L. S. T., Tan, J. X., Debby, E., & Aishworiya, R. (2021). Families with children with neurodevelopmental disorders during COVID-19: A scoping review. *Journal of Pediatric Psychology*, *46*(5), 514–525. 10.1093/jpepsy/jsab029 33758930 10.1093/jpepsy/jsab029 PMC8083717

[CR92] Skripkauskaite, S., Creswell, C., Shum, A., Pearcey, S., Lawrence, P., Dodd, H., & Waite, P. (2023). Changes in UK parental mental health symptoms over 10 months of the COVID-19 pandemic. *JCPP Advances*, *3*(2). 10.1002/jcv2.12139 10.1002/jcv2.12139 PMC10519732 37753154

[CR93] Spinelli, M., Lionetti, F., Pastore, M., & Fasolo, M. (2020). Parents’ stress and children’s psychological problems in families facing the COVID-19 outbreak in Italy. *Frontiers in Psychology*, *11*. 10.3389/fpsyg.2020.01713 10.3389/fpsyg.2020.01713 PMC7350926 32719646

[CR94] Spinelli, M., Lionetti, F., Setti, A., & Fasolo, M. (2020). Parenting stress during the COVID-19 outbreak: Socioeconomic and environmental risk factors and implications for children emotion regulation. *Family Process*, *60*(2), 639–653. 10.1111/famp.12601 32985703 10.1111/famp.12601

[CR95] Stewart, S. L., Babcock, S. E., & Dave, H. P. (2020). A psychometric evaluation of the interRAI Child and Youth Mental Health instruments (ChYMH) anxiety scale in children with and without developmental disabilities. *BMC Psychiatry*, *20*, 390. 10.1186/s12888-020-02785-9 32727428 10.1186/s12888-020-02785-9 PMC7390192

[CR96] Stewart, S. L., Celebre, A., Semovski, V., Hirdes, J. P., Vadeboncoeur, & Poss, J. W. (2022). The interRAI child and youth suite of mental health assessment instruments: An integrated approach to mental health service delivery. *Frontiers in Psychiatry*, *13*, 710569. 10.3389/fpsyt.2022.710569 35370860 10.3389/fpsyt.2022.710569 PMC8967950

[CR97] Stewart, S. L., & Hamza, C. (2017). The Child and Youth Mental Health Assessment (ChYMH): An examination of the psychometric properties of an integrated assessment developed for clinically referred children and youth. *BMC Health Services Research*, *17*, 82. 10.1186/s12913-016-1970-9 28122563 10.1186/s12913-016-1970-9 PMC5267403

[CR98] Stewart, S. L., Hirdes, J. P., Curtin-Telegdi, N., Perlman, C. M., McKnight, M., MacLeod, K., Ninan, A., Currie, M., Carson, S., Morris, J. N., Berg, K., Björkgren, M., Finne-Soveri, H., Fries, B. E., Frijters, D., Gray, L., Henrard, J-C., James, M. L., Ljunggren, G., Meehan, B., Smith, T. F., Steel, K., Szczerbińska, K., & Topinková, E. (2015). *interRAI Child and Youth Mental Health (ChYMH) Assessment Form and User’s Manual: For use with In-patient and Community-Based Assessments. Version 9.3.* interRAI.

[CR99] Stewart, S. L., LaRose, L., Gleason, K., Nicolson, R., McKnight, M., Knott, W., Currie, M., Hirdes, J. P., Curtin-Telegdi, N., Perlman, C. M., MacLeod, K., Ninan, A., Carson, S., Morris, J. N., Berg, K., Meehan, B., Björkgren, M., Fries, B. E., Frijters, D., Gray, L., Henrard, J-C., Hirdes, J., James, M., Ljunggren, G., Steel, K., & Topinková, E. (2015). *interRAI Child and Youth Mental Health and Developmental Disability (ChYMH-DD) Assessment Form and User’s Manual: For use with the inpatient and Community-Based Assessments. Version 9.3.* interRAI.

[CR100] Stewart, S. L., Toohey, A., & Poss, J. W. (2021). iCCareD: The development of an algorithm to identify factors associated with distress among caregivers of children and youth referred for mental health services. *Frontiers in Psychiatry*, *12*, 737966. 10.3389/fpsyt.2021.737966 34867533 10.3389/fpsyt.2021.737966 PMC8637612

[CR101] Sun, Y., Wu, Y., Fan, S., Dal Santo, T., Li, L., Jiang, X., Li, K., Wang, Y., Tasleem, A., Krishnan, A., He, C., Bonardi, O., Boruff, J. T., Rice, D. B., Markham, S., Levis, B., Azar, M., Thombs-Vite, I., Neupane, D., Agic, B., … Thombs, B. D. (2023). Comparison of mental health symptoms before and during the COVID-19 pandemic: Evidence from a systematic review and meta-analysis of 134 cohorts. *British Medical Journal, 380*, e074224. 10.1136/bmj-2022-074224 36889797 10.1136/bmj-2022-074224 PMC9992728

[CR102] Szpunar, M., Bourke, M., Vanderloo, L. M., Bruijns, B. A., Truelove, S., Burke, S. M., Gilliland, J., Irwin, J. D., & Tucker, P. (2023). Parent-reported changes in Ontario children’s physical activity levels during the COVID-19 pandemic. *Children*, *10*(2), 221. 10.3390/children10020221 36832350 10.3390/children10020221 PMC9954678

[CR103] Tawankanjanachot, N., Melville, C., Truesdale, M., & Kidd, L. (2024). An online survey of perspectives towards the impact of the COVID-19 pandemic amongst caregivers of adolescents with ASD. *BMC Nursing*, *23*(1), 830–815. 10.1186/s12912-024-02492-w 39543635 10.1186/s12912-024-02492-w PMC11566215

[CR104] Theall, L., Arbeau, K., Ninan, A., Willoughby, K., Ponti, M., Arnold, L., Dourova, N., & Currie, M. (2023). Caregiver and clinician experience with virtual services for children and youth with complex needs during COVID-19. *Journal of Pediatric Health Care*, *37*, 167–172. 10.1016/j.pedhc.2022.09.017 36307282 10.1016/j.pedhc.2022.09.017 PMC9534789

[CR105] Thomson, K. C., Jenkins, E., Gill, R., Richardson, C. G., Pettereni, M. G., McAuliffe, C., & Gadermann, A. M. (2021). Impacts of the COVID-19 pandemic on family mental health in Canada: Findings from a multi-round cross-sectional study. *International Journal of Environmental Research and Public Health*, *18*, 12080. 10.3390/ijerph182212080 34831830 10.3390/ijerph182212080 PMC8623196

[CR106] Toseeb, U. (2022). Sibling conflict during COVID-19 in families with special educational needs and disabilities. *British Journal of Educational Psychology*, *92*(1), 319–339. 10.1111/bjep.12451 34423422 10.1111/bjep.12451 PMC8646725

[CR107] Tso, W. W. Y., Wong, R. S., Tung, K. T. S., Nirmala, R., Fu, K. W., Yam, J. C. S., Chua, G. T., Chen, E. Y. H., Lee, T. M. C., Chan, S., K. W., Wong, W. H. S., Xiong, X., Chui, C. S., Li, X., Wong, K., Leung, C., Tsang, S. K. M., Chan, G. C. F., Tam, P. K. H., … Ip, P. (2020). Vulnerability and resilience in children during the COVID-19 pandemic. *European Child & Adolescent Psychiatry, 31*, 161–176. 10.1007/s00787-020-01680-8 33205284 10.1007/s00787-020-01680-8 PMC7671186

[CR108] UNICEF (2020). *Protecting the most vulnerable children from the impact of coronavirus: An agenda for action.* https://www.unicef.org/coronavirus/agenda-for-action

[CR109] Van den Steene, H., van West, D., & Glazemakers, I. (2019). Towards a definition of multiple and complex needs in children and youth: Delphi study in Flanders and international survey. *Scandinavian Journal of Child and Adolescent Psychiatry and Psychology*, *7*, 60–67. 10.21307/sjcapp-2019-009 33520769 10.21307/sjcapp-2019-009 PMC7709935

[CR110] Vella Fondacaro, D., Vella Fondacaro, F., & Camilleri, N. (2023). The impact of the COVID-19 pandemic on physical activity and anxiety levels of young mental health patients and their parents. *Sports Psychiatry*, *2*(2), 57–64. 10.1024/2674-0052/a000044

[CR111] Verdolini, N., Amoretti, S., Montejo, L., Garcia-Rizo, C., Hogg, B., Mezquida, G., Rabelo-da-Ponte, R., Valespir, C., Radua, J., Martinez-Aran, A., Pacchiarotti, I., Rosa, A. R., Bernardo, M., Vieta, E., Torrent, C., & Sole, B. (2021). Resilience and mental health during the COVID-19 pandemic. *Journal of Affective Disorders*, *283*, 156–164. 10.1016/j.jad.2021.01.055 33556749 10.1016/j.jad.2021.01.055 PMC7845537

[CR112] Visone, F., Di Gloria, S., Carlucci, L., & Gritti, P. (2022). Families in the storm: An observational study on family systems during the pandemic in an Italian sample. *Journal of Psychosocial Systems*, *11*(1), 6–9. 10.23823/jps.v6i1.98

[CR113] Wang, L., Zhang, H., Shang, C., Liang, H., Liu, W., Han, B., Xia, W., Zou, M., & Sun, C. (2022). Mental health issues in parents of children with autism spectrum disorder: A multi-time‐point study related to COVID‐19 pandemic. *Autism Research*, *15*(12), 2346–2358. 10.1002/aur.2836 36263600 10.1002/aur.2836 PMC9874755

[CR114] Westrupp, E. M., Bennett, C., Berkowitz, T., Youssef, G. J., Toumbourou, J. W., Tucker, R., Andrews, F. J., Evans, S., Teague, S. J., Karantzas, G. C., Melvin, G. M., Olsson, C., Macdonald, J. A., Greenwood, C. J., Mikocka-Walus, A., Hutchinson, D., Fuller-Tyszkiewicz, M., Stokes, M. A., Olive, L., Wood, A. G., … Sciberras, E. (2023). Child, parent, and family mental health and functioning in Australia during COVID-19: Comparison to pre-pandemic data. *European Child & Adolescent Psychiatry*, *32*, 317–330. 10.1007/s00787-021-01861-z 34417875 10.1007/s00787-021-01861-z PMC8379590

[CR115] Willner, P., Rose, J., Kroese, B. S., Murphy, G. H., Langdon, P. E., Clifford, C., Hutchings, H., Watkins, A., Hiles, S., & Cooper, V. (2020). Effect of the COVID-19 pandemic on the mental health of carers of people with intellectual disabilities. *Journal of Applied Research in Intellectual Disabilities*, *33*, 1523–1533. 10.1111/jar.12811 32885897 10.1111/jar.12811

[CR116] Wolstencroft, J., Hull, L., Warner, L., Akhtar, T.N., Mandy, W., IMAGINE-ID consortium, & Skuse, D. (2021).'We have been in lockdown since he was born’: A mixed methods exploration of the experiences of families caring for children with intellectual disability during the COVID-19 pandemic in the UK. *British Medical Journal Open*, *11*, e049386. 10.1136/bmjopen-2021-049386 10.1136/bmjopen-2021-049386 PMC8487017 34593495

[CR117] World Health Organization (2020, April 27). *WHO timeline – COVID-19.* https://www.who.int/news-room/detail/27-04-2020-who-timeline---covid-19

[CR118] Zampini, L., Zanchi, P., Riva, P., & Tobia, V. (2024). Associations between social isolation and parenting stress during the first wave of COVID-19 in Italian children with special educational needs. *International Journal of Developmental Disabilities*. 10.1080/20473869.2022.2062841 10.1080/20473869.2022.2062841 PMC10916901 38456135

