# Correction to: Families of Children/Youth with Complex Needs Before, During, and After COVID-19 Pandemic Restrictions

**DOI:** 10.1007/s40653-025-00685-2

**Published:** 2025-01-24

**Authors:** Kim Arbeau, Serena Atallah, Jeff St Pierre

**Affiliations:** 1 Applied Research and Evaluation Department, Child and Parent Resource Institute, 600 Sanatorium Road, London, ON N6H 3W7 Canada; 2 Department of Psychology, Social Science Centre, Western University, London, ON N6A 5C2 Canada

**Correction to: Journal of Child and Adolescent Trauma**

10.1007/s40653-024-00676-9

The original online version of this article has been revised. The third author name is incorrect in this citation: Arbeau, K., Atallah, S. & Pierre, J.S. Families of Children/Youth with Complex Needs Before, During, and After COVID-19 Pandemic Restrictions. Journ Child Adol Trauma (2025). 10.1007/s40653-024-00676-9.

Here is the corrected citation for Dr. St. Pierre’s authorship:

Arbeau, K., Atallah, S. & St. Pierre, J. Families of Children/Youth with Complex Needs Before, During, and After COVID-19 Pandemic Restrictions. Journ Child Adol Trauma (2025). 10.1007/s40653-024-00676-9.

